# Acute lysine supplementation does not improve hepatic or peripheral insulin sensitivity in older, overweight individuals

**DOI:** 10.1186/1743-7075-11-49

**Published:** 2014-10-08

**Authors:** Il-Young Kim, Rick H Williams, Scott E Schutzler, Cosby J Lasley, Donald L Bodenner, Robert R Wolfe, Robert H Coker

**Affiliations:** Center for Translational Research in Aging and Longevity, Reynolds Institute on Aging, University of Arkansas for Medical Sciences, Little Rock, AR USA; Center for Alaska Native Health Research, Institute of Arctic Biology, University of Alaska-Fairbanks, 902 North Koyukuk Drive, Fairbanks, AK 99775-7000 USA

**Keywords:** Insulin resistance, Liver, Muscle

## Abstract

**Context:**

Lysine supplementation may have a positive influence on the regulation of glucose metabolism but it has not been tested in the geriatric population. Objective: We evaluated the impact of acute lysine supplementation using three randomized experimental scenarios: 1) oral glucose alone (control), 2) oral glucose and low-dose lysine (2 grams), and oral glucose and high dose lysine (5 grams) lysine in 7 older (66 ± 1 years/age), overweight/obese (BMI = 28 ± 2 kg/m^2^) individuals.

**Methods:**

We utilized a dual tracer technique (i.e., [6,6-^2^H_2_] glucose primed constant infusion and 1-[^13^C] glucose oral ingestion) during an oral glucose tolerance test (OGTT) to examine differences in hepatic and peripheral insulin sensitivity under all three scenarios.

**Results:**

Post-absorptive plasma glucose and insulin concentrations were not different between the three trials. Similarly, the response of glucose and insulin concentrations during the oral glucose tolerance tests (OGTT) was similar in the three trials. The results of the Matsuda index (ISI/M) were also not different between the three trials. As an index of hepatic insulin sensitivity, there were no significant differences in the endogenous glucose rate of appearance (glucose R_a_) for control, 2 g lysine and 5 g lysine (1.2 ± 0.1, 1.1 ± 0.1, 1.3 ± 0.1 mg•kg^-1^•min^-1^), respectively. With respect to peripheral insulin sensitivity, there were no significant differences in the glucose rate of disappearance (glucose R_d_) for control, 2 g lysine and 5 g lysine (4.2 ± 0.1, 4.3 ± 0.2, and 4.5 ± 0.4 mg•kg^-1^•min^-1^), respectively.

**Conclusions:**

Previous studies in younger participants have suggested that lysine may have a beneficial effect on glucose metabolism. However, acute lysine supplementation in the older population does not facilitate beneficial changes in glucose R_a_ or glucose R_d_.

**Electronic supplementary material:**

The online version of this article (doi:10.1186/1743-7075-11-49) contains supplementary material, which is available to authorized users.

## Introduction

Cardiovascular disease (CVD) remains a leading cause of death among US adults [1]. Elderly individuals are at high risk for negative cardiovascular outcomes due to age-related physiological changes that directly impact the anatomy and function of the cardiovascular system [2, 3], as well as disease-related processes that are driven by poor nutritional status, physical inactivity, and other issues common at older ages [4, 5]. In addition, the accelerated loss of muscle mass (sarcopenia) that is typical among elderly adults, combined with the increasing prevalence of obesity in this population [6] present additional difficulties when choosing an appropriate therapeutic course of action in this population.

Hyperglycemia is one important factor that directly influences vascular complications associated with CVD and represents a central therapeutic target for managing CVD-related outcomes in elderly adults. Although recent trials have demonstrated that intensive glucose control may be harmful compared to less-intensive standard treatment in older patients with advanced type 2 diabetes [7, 8], tight glycemic control was associated with slowed progression of retinopathy [9, 10] and may especially reduce the risk of micro- and macrovascular disease when started earlier in the disease course [11]. The ingestion of protein with glucose has been shown to augment insulin secretion and diminish the plasma glucose response in persons with type 2 diabetes [12]. It has been recently reported that lysine (a readily abundant essential amino acid), when ingested with glucose, facilitates a reduction in the glucose area under the curve (AUC) by 44% without any change in plasma insulin, along with a reduction in plasma glucagon [13]. Thus, lysine supplementation may represent a novel and cost-effective therapeutic intervention to reduce hyperglycemia and related CVD outcomes without potential complications associated with other nutritional, behavioral, or pharmacological interventions.

It is not known whether acute lysine supplementation in the previously mentioned study [13] promoted improved glucose metabolism through 1) an increased suppression of endogenous glucose production (glucose R_a_), 2) increased glucose disappearance (glucose R_d_), or 3) both. Second, since almost all of the study subjects experienced some sort of mild gastrointestinal distress with higher doses of lysine, the efficacy of a lower dose of lysine requires investigation. In order to validate these preliminary studies and investigate the site of action, we propose to use a dual tracer technique ([1-^13^C] glucose oral ingestion and [6,6-^2^H_2_] glucose primed constant infusion) that will allow us to selectively delineate endogenous glucose R_a_, exogenous glucose R_a_, and endogenous glucose R_d_ [14]. In this way, we will be able to determine the primary mechanism (increased suppression of glucose R_a_, improved glucose R_d_ or both) responsible for the improvement in glucose AUC.

Therefore, the primary objective of this study was to determine the efficacy of acute lysine supplementation on glucose metabolism in older, overweight individuals. In addition, we incorporated the application of stable isotope tracer methodology coupled with an OGTT that allowed us to determine the site of action (hepatic and/or peripheral) that was influenced by lysine supplementation. To our knowledge, the influence of lysine supplementation on hepatic and peripheral glucose metabolism when given in combination with an OGTT has not been investigated. The practical implications of a readily available, efficacious nutrient that could be used to promote more effective glucose homeostasis have important implications.

## Methods

### Subjects

Seven overweight/obese older Caucasian subjects (66 ± 4 yr, 4 M, 3 F, 175 ± 7 cm, 98.3 ± 9.6 kg, body mass index = 32 ± 2.2 kg^●^m^-2^; % fat = 33.6 ± 3.4, means ± SD) participated. All subjects were weight stable, and ranged between normal glucose tolerance (NGT) and impaired glucose tolerance (IGT). Participants underwent a physical examination, which included a medical history, blood chemistry, measurement of vital signs and body weight (within 0.1 kg). A resting electrocardiogram (EKG) and dual X-ray absorptiometry (DXA) scan were also performed at baseline. Participants taking medicines or supplements that might have had potential effects on metabolism were excluded. Each participant provided written informed consent, and study procedures were approved by the Institutional Review Board of the University of Arkansas for Medical Sciences (UAMS).

### Study design

The order of the visits was randomly assigned to the three supplements 1) glucose alone or control, 2) glucose + low dose and 2 g lysine (2 L), and 3) glucose + high dose or 5 g lysine (5 L). The research staff administered these supplements in conjunction with a dual tracer technique (i.e., [1-^13^C]glucose oral ingestion and [6,6-^2^H_2_] glucose primed constant infusion) during an oral glucose tolerance test (OGTT-DT) on three separate occasions with a 2 week wash out period between each metabolic study. The supplements provided were identical in appearance. The [6,6-^2^H_2_] glucose tracer was primed constantly infused [prime, 82.2 umol/kg; rate, 0.78 umol•kg^-1^•min^-1^]. Ingestion of 1-[^13^C] glucose was concurrent with the oral glucose test drink +/- lysine supplement and was based on 40 mg/kg (15). Randomization to the order of the study visits was determined prior to enrollment.

**Figure 1:**
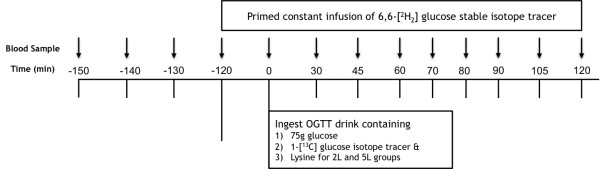
**Isotope tracer infusion protocol.**

Blood samples (t = -150, -140, and -130 min) were collected prior to the onset of the OGTT-DT protocol, and serial blood samples (t = 30, 45, 60, 70, 80, 90, 105, and 120 min) were collected during the remainder of the study into EDTA-containing tubes and centrifuged at 3,500 rpm for 15 min at 4°C. Oral glucose was provided as Sundex ® (Thermo Fisher Scientific, Waltham, MA) and lysine was administered as L-lysine dissolved into deionized water at a concentration of 50 g/100 mL (Ajinomoto USA Inc., Raleigh, NC) (Figure 1). Stable isotopes were obtained as sterile powders (Cambridge Isotope Labs, Andover, MA), and were professionally prepared under aseptic conditions by a Research Pharmacist at UAMS. The dual tracer technique utilized the simultaneous infusion and ingestion of glucose tracers to trace the metabolic fate of ingested and endogenously-produced glucose. Our primary endpoints were glucose R_a_ and glucose R_d_ (Figure 1). This approach allowed us to determine the variable impact of lysine co-ingestion with glucose on the hepatic and peripheral mechanisms that may be responsible for potential improvements in glycemia.

### Calculations

Whole-body insulin sensitivity was estimated by the Matsuda Insulin Sensitivity Index (ISI/M) = 10000/square root of ([fasting glucose × fasting insulin] × [mean glucose × mean insulin during OGTT]) [15]. We calculated glucose AUC and insulin AUC using the trapezoidal rule applied to the insulin and glucose responses during the OGTT [16]. Insulin sensitivity in the post-absorptive state was evaluated using the homeostasis model assessment (HOMA-IR) index that is calculated by dividing the product of fasting plasma glucose and fasting plasma insulin by 22.5 [17]. Calculation of the insulinogenic index was completed using the equation: (Ins_30_ –Ins_0_/Glucose_30_ Glucose_0_). The disposition index was calculated as the product of the Matsuda index and the insulinogenic index [18].

For calculation of glucose kinetics the Steele equation was used for the non-steady state [19]. This approach has been widely discussed for at least 50 years. The major issue with regard to calculating glucose kinetics with the Steele equation is that a factor is used to account for the fact that the glucose pool does not behave as a single, rapidly-mixing pool. The assumption underlying the correction factor is that there is some fraction of the total glucose pool that acts as a single, well-mixed pool in the acute, non-steady state circumstance. We have used different approaches, both experimental and theoretical, to investigate the correct volume of distribution for use with the Steele equation for glucose kinetics [20, 21]. The conclusion from these studies was that there is no unique value that results in the correct calculation of glucose R_a_ in all circumstances. Glucose R_a_ is relatively accurately calculated during acute changes in isotopic enrichment when a relatively small value approximating plasma volume (40 ml/kg) is used. As a new isotopic equilibrium is approached, changes in plasma enrichment of glucose reflect a volume of distribution significantly greater than the plasma volume, with a value as large as 200 ml/kg being optimal. It is impossible to anticipate a priori the most appropriate value at any given time. Rather, it is necessary to choose a single value and use that value throughout the entire experiment. It has been our practice to use a small (40 ml/kg) value for the reason that the acute responses are consistently more accurately predicted, and the fact that the importance of the exact volume used becomes less important as a new isotopic equilibrium is approached. This is because the difference in isotopic enrichment between samples diminishes as a new equilibrium is approached, so the volume is multiplied by a diminishingly small number [22]. In the current study, we calculated glucose R_a_ using values of 40 ml/kg and 200 ml/kg to estimate the upper and lower bounds of glucose R_a_ in the non-steady state. As predicted from our earlier work, in every case the magnitude of the acute change in glucose R_a_ from the basal value was markedly greater when 200 ml/kg was used, and as a new equilibrium was approached the values became similar regardless of the volume used in the calculation. We have presented in the current paper only the results from the calculation using a value of 40 ml/kg, because the initial changes in glucose R_a_ calculated using 200 ml/kg were likely overestimates of the true response [22]. Use of different volumes did not affect our conclusions, only the magnitude of the early response.

Plasma glucose tracer enrichments and concentrations were curve-fitted with a 3-order polynomial model over the OGTT period in Graphpad Prism 5 for Mac (Graphpad Software, Inc. La Jolla CA). Enrichment (E) is expressed as mole percent excess (MPE): MPE is calculated as TTR/(1 + TTR), where TTR is tracer to tracee ratio. Appropriate corrections for skew abundance distribution and overlapping spectra for TTR of 1-[^13^C] gluscoe and 6,6-[^2^H_2_] glucose were made, respectively [22]. From these calculations, total glucose R_a_ is comprised of ingested glucose, hepatic glucose production, and negligible renal glucose production or splanchnic glucose R_a_:

Total glucose R_a_ = (F – (p*V* • (C_2_ + C_1_) / 2) • ((E_2_ – E_1_) / (t_2_ – t_1_))) / (E_2_ + E_1_) / 2 [1].

Glucose R_d_ = Total glucose R_a_ – p*V* • (C_2_ – C_1_) / (t_2_ – t_1_) [2].

Exogenous glucose Ra = Total glucose Ra • (E_P_ / E_D_) [3].

Endogenous glucose Ra = Total glucose Ra – Exogenous glucose Ra [4].

MCR = Glucose Rd / ((C_1_ + C_2_) / 2) [5].

Where F represents the infusion rate of 6,6-[^2^H_2_] glucose; p*V* is the effective volume of distribution for glucose, for which 40 ml•kg^-1^ was used; C_1_ and C_2_ are plasma glucose concentrations at specific times t_1_ and t_2,_ respectively, E_1_ and E_2_ are plasma enrichment of 6,6-[^2^H_2_] glucose at specific times t_1_ and t_2,_ respectively; E_D_ and E_P_ are enrichments of 1-[^13^C] glucose from the test drink and plasma, respectively.

### Data handling and recordkeeping

All data and research material were obtained specifically for research purposes and were made available only to the medical staff and the principal investigator or his associates. These data included demographic data, body composition data, multiple blood samples, glucose metabolic testing data, screening laboratory data, and a full medical history.

The UAMS pharmacy performed the randomization; repackaging and relabeling of supplements. The supplements were similar in appearance and labeled with the pre-selected randomization codes. Randomization codes were saved at the Institute on Aging in a sealed envelope. The seal was broken at the end of the study; and after the blood samples had been analyzed.

### Statistical and power analyses

An analysis of covariance model was employed to assess the effects of lysine intake (5 and 2 g/day) on hepatic and peripheral glucose metabolism in older, obese participants. Two levels of acute lysine intake were included as factors in the model, while hepatic and glucose metabolism were included as covariates.

Initially, a lysine-by-glucose metabolism interaction term was included in the model and tested. If the interaction term was significant, then the effect of lysine intake on glucose metabolism was tested separately for hepatic and peripheral tissue. A 5% α-level was used to determine statistical significance for tests of main effects, while a 10% α-level was used to test the interaction effect.

## Results

Additional file 1: Table S1: Clinical Characteristics.

Glucose. Fasting plasma glucose (112 ± 5, 111 ± 5 and 112 ± 5 mg/dl for the conditions of the control, 2 L and 5 L, respectively) represented impaired glucose tolerance and were not significantly different. Plasma glucose increased (*P* < 0.0001), and in a similar fashion (*P* = 0.91) in all three experimental scenarios during the OGTT-DT (Figure 1). Glucose AUC was similar (*P* = 0.86) at 19247 ± 1794, 17617 ± 1088 and 17193 ± 1175 mg/dl^●^120 min for the control, 2 L and 5 L during the OGTT-DT.

**Figure 2:**
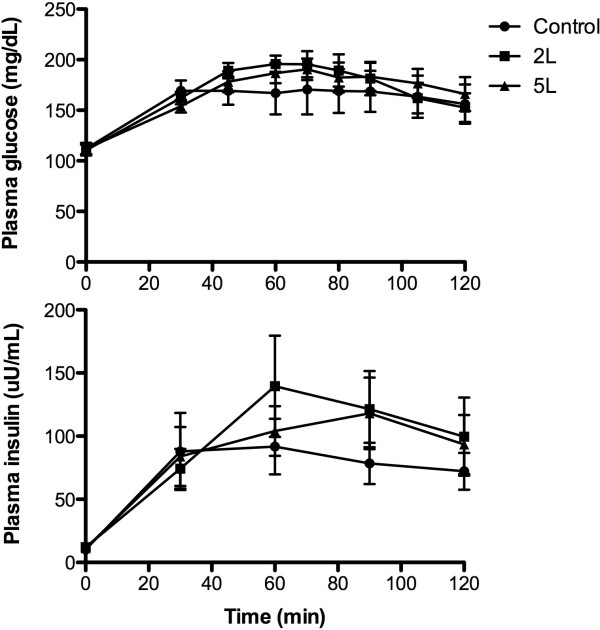
**Plasma concentrations of glucose and insulin during the OGTT-DT in the control, 2 grams lysine, and 5 grams of lysine.** Data are mean ± SEM. Significant increase over time for all three groups (*P* < 0.05).

Plasma Insulin. Fasting plasma insulin was 10.6 ± 1.5, 12.0 ± 1.7 and 11.3 ± 1.0 uU/ml in the control, 2 L and 5 L conditions, respectively, and increased (*P* < 0.0001) without any significant difference (*P* = 0.71) between the conditions during the OGTT-DT (Figure 2). Insulin AUC was similar (*P* = 0.72) at 8979 ± 2020, 11730 ± 2864, and 10573 ± 2226 uU/ml ≅ 120 min for the control, 2 L and 5 L, respectively.

Insulin Sensitivity. The HOMA-IR index was 2.6 ± 0.3, 3.4 ± 0.6, and 3.2 ± 0.4, and was similar (*P* = 0.44) for the control, 2 L and 5 L. Insulin sensitivity calculated from the ISI/M was similar (*P* = 0.86) at 3.3 ± 0.6, 3.0 ± 0.6 and 2.9 ± 0.5 for control, 2 L and 5 L, respectively.

Insulinogenic and Disposition Index. With respect to differences in peripheral changes in insulin and glucose during the OGTT, there were no significant differences based on the insulinogenic index (1.4 ± 0.4, 1.3 ± 0.43, and 2.0 ± 0.7) (*p* = 0.6598) between control, 2 L, and 5 L, respectively. There were also no significant differences in the disposition index (3.8 ± 0.9, 3.3 ± 0.4, and 1.7 ± 0.2) (*P* = 0.08), respectively.

**Figure 3:**
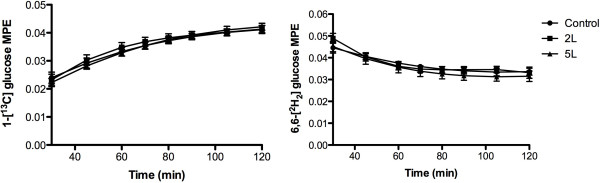
**Plasma enrichment, expressed as mole percent excess (MPE), for 1-[** ^**13**^ **C] glucose and 6,6-[** ^**2**^ **H**_**2**_ **] glucose during the OGTT-DT in the control, 2 grams lysine, and 5 grams of lysine.** Data are mean ± SEM. Significant decrease over time for all three groups (*P* < 0.05).

Glucose Enrichments. Initially, the 6,6-^2^[H_2_] glucose MPE was similar (*P* = 0.68) and decreased (*P* < 0.0001) without any significant variation between the control, 2 L or 5 L (Figure 3). The 1-[^13^C] MPE was also similar at baseline (*P* = 0.91), but increased similarly in the control, 2 L and 5 L (Figure 3).

**Figure 4:**
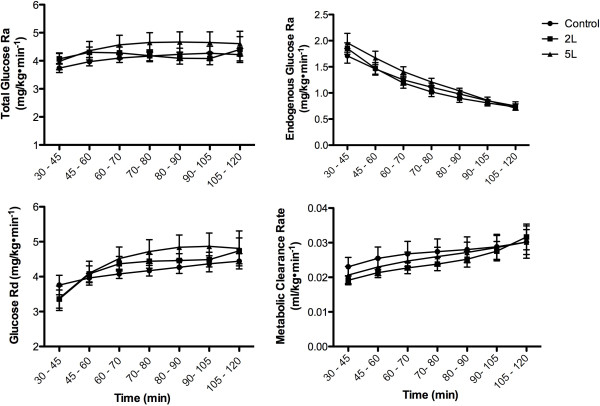
**Glucose kinetics during the OGTT-DT in the control, 2 grams lysine, and 5 grams of lysine.** Data are mean ± SEM. Significant increase over time for all three groups (*P* < 0.05).

Glucose Kinetics. Total Glucose R_a_ was not different (*P* = 0.49) between the control, 2 L and 5 L during the OGTT-DT (Figure 4). Exogenous glucose R_a_ increased (*P* < 0.0001) during the OGTT-DT and was not different (*P* = 0.63) between the control, 2 L and 5 L. Endogenous glucose R_a_ decreased (*P* < 0.0001) in the control (1.7 ± 0.1 to 0.8 ± 0.1 mg•kg^-1^•min^-1^), 2 L (1.9 ± 0.1 to 0.7 ± 0.1 mg•kg^-1^•min^-1^) and 5 L (1.9 ± 0.7 to 0.7 ± 0.1 mg•kg^-1^•min^-1^) and was not different between groups (Figure 4). Glucose R_d_ increased (*P* < 0.0001) in a similar fashion (*P* = 0.69) in the control (3.8 ± 0.3 to 4.4 ± 0.2 mg•kg^-1^•min^-1^), 2 L (3.4 ± 0.3 to 4.7 ± 0.4 mg•kg^-1^•min^-1^) and 5 L (3.4 ± 0.3 to 4.9 ± 0.4 mg•kg^-1^•min^-1^) (Figure 4). Independent of the differences in glucose concentration, the metabolic clearance rate increased in a similar fashion (*P* = 0.69) in the control (2.3 ± 0.3 to 3.0 ± 0.4 ml/kg•min^-1^), 2 L (1.9 ± 0.3 to 3.2 ± 0.4 ml/kg•min^-1^) and 5 L (2.1 ± 0.3 to 3.0 ± 0.5 ml/kg•min^-1^) (Figure 4).

## Discussion

Previous studies have suggested the potential efficacy of acute lysine supplementation on glucose metabolism. In an effort to clearly delineate the potential mechanisms responsible for lysine-induced improvements in glucose metabolism, we utilized a dual tracer ([6,6-^2^H_2_] glucose primed constant infusion and [1-^13^C]glucose oral ingestion) during an oral glucose tolerance test in older, overweight individuals. Instead of relying solely on glucose AUC, HOMA-IR, or ISI that may have interpretative limitations in acute or intervention-based studies [16], this dual tracer approach allowed us to clearly delineate glucose kinetics during OGTT, which is commonly utilized in the clinical setting. Nonetheless, the data generated from all methods were uniformly consistent, and our results were not consistent with our original hypotheses. In fact, we found no significant differences in glucose AUC, HOMA-IR or ISI between lysine supplementation and control. More specifically, lysine supplementation had no influence endogenous glucose R_a_ (reflective of hepatic insulin sensitivity) or glucose R_d_ (indicative of peripheral insulin sensitivity). The MCR of glucose was also similar in the control, 2 L and 5 L, highlighting the lack of any difference in the amount of lysine ingestion on glucose kinetics.

Our results were a bit surprising given that previous studies utilizing lysine supplementation in younger, healthy individuals described beneficial changes in the glucose AUC [13]. While it has been known for almost 50 years that amino acids promote the release of insulin [23], the positive results of the previous lysine supplementation studies occurred without a difference in peripheral insulin during the OGTT [13]. In another previous study utilizing a combination of arginine/lysine, there was no influence of plasma insulin, glucose or glucose AUC [24]. This is significant for many reasons, including the well-established relationship between the infusion or ingestion of amino acids and the insulinotrophic response that mediates potentially beneficial effects on glucose metabolism [23]. Even in the earlier studies that demonstrated positive results on glucose metabolism, wide variations were described among essential amino acids with regard to the release of insulin [23]. We found that acute lysine supplementation did not seem to stimulate insulin secretion as reflected by almost identical plasma insulin levels in all three conditions, and this corroborates the findings of the most recent study involving lysine supplementation in younger individuals [13]. On the other hand, we did not measure C-peptide in the present study and this precludes us from knowing whether differences in hepatic clearance contributed to relatively equivalent peripheral insulin concentrations. The discrepancy in the plasma insulin response is most likely due to 8–10 fold differences in the amount of amino acid ingested in the different studies. With ingestion of a modest amount of lysine there is no response of plasma insulin and consequently no effect on glucose metabolism. Further, since the study population in the present study were older, overweight/obese adults with lower doses of lysine (2 or 5 g of lysine) as compared to healthy young adults with a wide range of BMI (21 – 39 kg^●^m^-2^) in the previous study with higher doses of lysine (mean, 11 g; range, 6 – 13 g) [13], the null effect of acute lysine supplementation on glucose metabolism in the present study can be attributed to two factors: 1) doses of lysine ingestion and 2) age. First, the lower lysine doses in the present study may be simply insufficient to produce a favorable effect on glucose metabolism. Second, there may be an age-associated resistance to a lower dose of lysine ingestion with respect to glucose metabolism as in the case for muscle protein synthesis response. It has been shown that with an advancing age, muscle protein synthesis (MPS) response to a smaller ingestion of EAAs (7 g) was reduced in older individuals as compared to young individuals [25], which was overcome by a larger ingestion of EAAs (15 g) [26]. Therefore, it is worth investigating whether ingestion of lower doses of lysine as in the case of the present study could improve glucose metabolism in healthy young adults.

The dual tracer method in the context of OGTT is not a new technique, and has been used extensively to evaluate potential changes in glucose metabolism [14, 27]. Inappropriate estimation of the effective volume of distribution for glucose can lead to erroneous variations including the almost complete suppression of endogenous glucose R_a_ in overweight individuals with insulin resistance [28]. In the present study, endogenous glucose R_a_ decreased gradually and consistently in all three conditions without consistent and subtle variations in [6,6-^2^H_2_]glucose and [1-^13^C]glucose enrichments. In addition, endogenous glucose R_a_ increased as anticipated throughout the OGTT with any variation between the three conditions. Our approach was further strengthened by the randomized design utilizing the same participants.

The influence of glycine [29], proline [30], arginine [31], phenylalanine [32], and leucine [33] on glucose metabolism has been evaluated. Of these, leucine and phenylalanine seem to promote efficacious changes in glucose metabolism [32, 33]. Despite very few studies to support its efficacy, lysine supplements are often marketed to improve the regulation of glucose metabolism in diabetes and metabolic syndrome without sufficient clinical evidence. This may be largely based on the contention that the co-ingestion of protein with glucose has a synergistic influence on the release of insulin [12]. In so far as lysine itself is concerned, it only seems to function as a powerful insulin secretagogue when ingested in higher amounts (i.e., >11 grams) or in the context of a high protein meal [12]. One of the reasons that we chose to evaluate responses to 2 and 5 grams of lysine were due to previous reports of gastrointestinal distress, primarily diarrhea, with higher doses. This would contraindicate the use of lysine in older adults. Notably, there were no reports of gastrointestinal distress in the current study.

We evaluated the influence of acute lysine supplementation on glucose metabolism using a randomized experimental design in the same subjects. We found that acute lysine ingestion at moderate doses (i.e., 2 or 5 g) has no favorable influence on endogenous glucose R_a_, endogenous glucose R_d_, glucose AUC, insulin AUC or ISI/M in older, overweight individuals. Given that larger doses may be required, future studies are needed to evaluate whether the risk of gastrointestinal distress might outweigh the therapeutic benefit of lysine ingestion.

## Electronic supplementary material

Additional file 1: Table S1: Clinical Characteristics. (DOCX 20 KB)
