# Supplementary material for: Acute lysine supplementation does not improve hepatic or peripheral insulin sensitivity in older, overweight individuals
Source: Nutr Metab (Lond). 2014 Oct 8;11:49. doi: 10.1186/1743-7075-11-49 (PMC4198625; doi:10.1186/1743-7075-11-49)
Supplement: Supplementary file 1 — Additional file 1: Table S1: Clinical Characteristics. (DOCX 20 KB) [file 12986_2014_620_MOESM1_ESM.docx]

|  | Age | Body weight (kg) | Height (cm) | Body mass index (kg/m^2^) | Fat Free Mass | % Fat |
| --- | --- | --- | --- | --- | --- | --- |
| Mean±SEM | 66.4±1.3 | 98.3±3.6 | 175.1±2.7 | 32±0.8 | 60.1±2.8 | 33.5±1.3 |

Additional file 1: Table S1. Clinical Characteristics
